# Segmented arch or continuous arch technique? A rational approach

**DOI:** 10.1590/2176-9451.19.2.126-141.sar

**Published:** 2014

**Authors:** Sergei Godeiro Fernandes Rabelo Caldas, Alexandre Antonio Ribeiro, Hallissa Simplício, André Wilson Machado

**Affiliations:** 1 Adjunct professor, Department of Pediatric Dentistry, Federal University of Rio Grande do Norte (UFRN), professor at the Specialization course of Orthodontics, UnP.; 2 PhD resident in Orthodontics, State University of São Paulo (UNESP)/ Araraquara. Professor.; 3 Adjunct professor, Department of Pediatric Dentistry and Orthodontics, UFRN.; 4 Adjunct professor, Department of Orthodontics, Federal University of Bahia (UFBA). Visiting professor, Master's program in Orthodontics, UCLA.

**Keywords:** Orthodontics, Corrective Orthodontics, Biomechanics

## Abstract

This study aims at revising the biomechanical principles of the segmented archwire technique as well as describing the clinical conditions in which the rational use of scientific biomechanics is essential to optimize orthodontic treatment and reduce the side effects produced by the straight wire technique.

## Abstract (pt)

O objetivo desse trabalho é revisar os princípios biomecânicos da técnica do arco segmentado, bem como descrever situações clínicas onde o uso racional da biomecânica científica é fundamental na otimização do tratamento ortodôntico e eliminação dos efeitos colaterais da abordagem com arco contínuo.

## INTRODUCTION

Specialists in Orthodontics, particularly beginners, often have many questions in mind: Which technique should be used? What is the best bracket prescription? Which is the most recommendable type of bracket: conventional or self-ligating? When are the straight archwire and segmented mechanics techniques best recommended?

**Figure 1:**
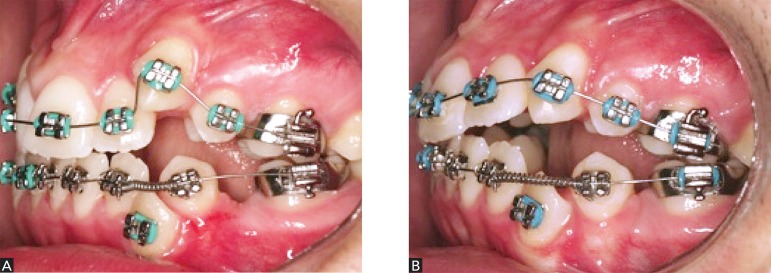
**A**) Straight wire mechanics used for canine extrusion. **B**) Note the side effects on the lateral incisor and first premolar, which made the conditions of the case worse.

Making an accurate diagnosis of the changes observed in patients is necessary. Likewise, an effective treatment plan performed within a shorter period of time, with as little injuries to protective and supporting tissues as possible, is also essential. Thus, regardless of the type of bracket, prescription, technique or slot, recommending the most appropriate treatment is what really matters. For this reason, knowing the principles on which each technique is based on, as well as its limitations, is essential. For instance, treating a clinical case of a maxillary canine in infralabioversion by means of the straight archwire technique used to level the tooth is a harmful procedure for adjacent teeth. Canine extrusion would occur regardless of the type of bracket, whether conventional or self-ligating, however, it would be followed by undesired intrusion and failure of the lateral incisor and first premolar (Fig 1). Many authors claim that such undesired effect would not result in any further issues, given that it would be solved with intermaxillary rubber bands, arch bends or wire progression. However, it results in a side effect of which solution demands extra treatment time, results in increased biological damage and potential root resorption. Thus, in this case, it is incongruous to use straight wires to level a canine, regardless of the type of bracket.

**Figure 2:**
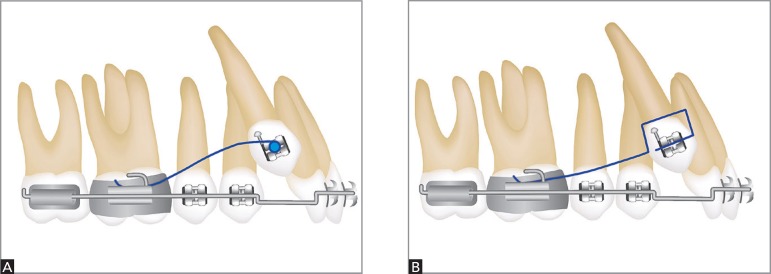
Cantilever (**A**) and rectangular loop (**B**) for canine extrusion.

Conversely, a complementary technique can be used to employ such procedure without producing further side effects on adjacent teeth. With the aid of the segmented arch technique (SAT) and after healing of the anchorage unit, only the canine is extracted by a cantilever or a rectangular loop (Fig 2). Differently from the conventional techniques, which normally use an arch made of one single alloy, connecting all brackets and adjacent tubes; the SAT uses arch segments connected to each other, but not necessarily connected to brackets and adjacent tubes. This allows a combination of wires made of different alloys, dimensions and hardness to be used. Rigid and thick archwires can connect groups of teeth into anchorage units, whereas flexible archwires are used to exert forces between these units.^1^

After employing the segmented mechanics, treatment can be conventionally performed with straight wires. Thus, this article aims at discussing how SAT can aid orthodontists to solve specific cases by means of a rational approach.

## PRINCIPLES BEHIND THE SEGMENTED ARCH TECHNIQUE

SAT was developed by Dr. Charles Burstone in 1962. It consists of a sequence of orthodontic procedures based on the mechanical principles of Mechanics. Thus, in order to fully understand the principles behind it, it is essential to comprehend the following concepts: horizontal force, torque, moment of force, moment of torque, equivalent system of forces, moment-to-force ratio and load/deflection ratio.^2^

Horizontal force is a vector able to overcome inertia, thus changing the position of the moving body. Torque refers to two noncollinear, coplanar forces of same intensity, but opposite direction. A body subjected to torque tends to rotate about an axis, in which the center of resistance (CRes) coincides with the center of rotation (CRot), causing a tooth to go through pure rotation.^1,2,3^

**Figure 3:**
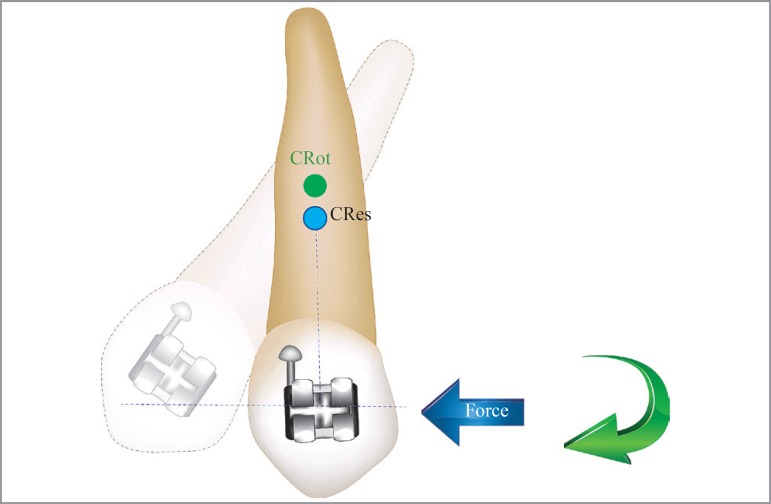
Uncontrolled tipping with force bypassing the CRes. Note that the CRot is apically displaced. (Source: adapted from Almeida^31^).

Nevertheless, whenever a horizontal force is applied to a tooth and this force bypasses the CRes, the latter produces a rotational movement known as "moment of force". In this case, the crown moves in one direction, while the root moves in the opposite direction, with the CRot more apical towards the center of resistance. This movement is known as uncontrolled tipping (Fig 3).^1,2,3^

**Figure 4:**
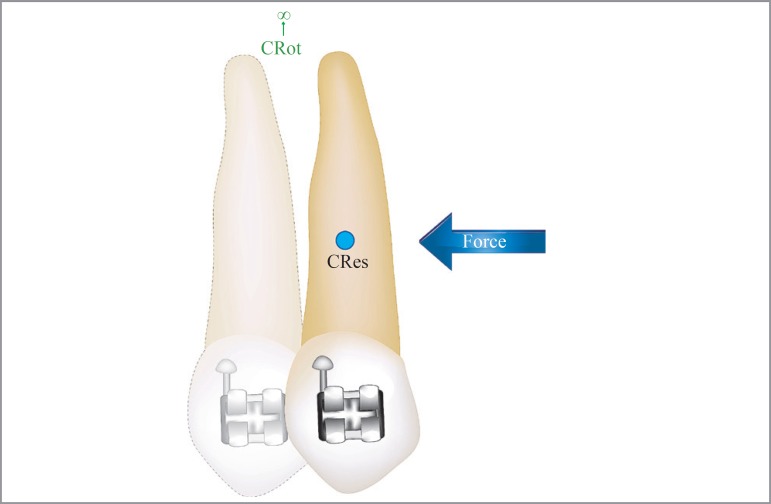
Translatory movement with force going exactly through the CRes. Note that the CRot is in the infinite. (Source: adapted from Almeida^31^).

On the other hand, should the force go exactly through the CRes, this tooth will move by translation (Fig 4), in which case the CRot is in the infinite. However, it is worth mentioning that it is extremely difficult to have force going exactly through the CRes. Therefore, in order to achieve a translatory movement, it is important to establish an equivalent system of forces that is applicable to a bracket. In other words, a different system of forces that produces the same effect on the CRes of a tooth, even if applied to the crown.^1,2,3^

**Figure 5:**
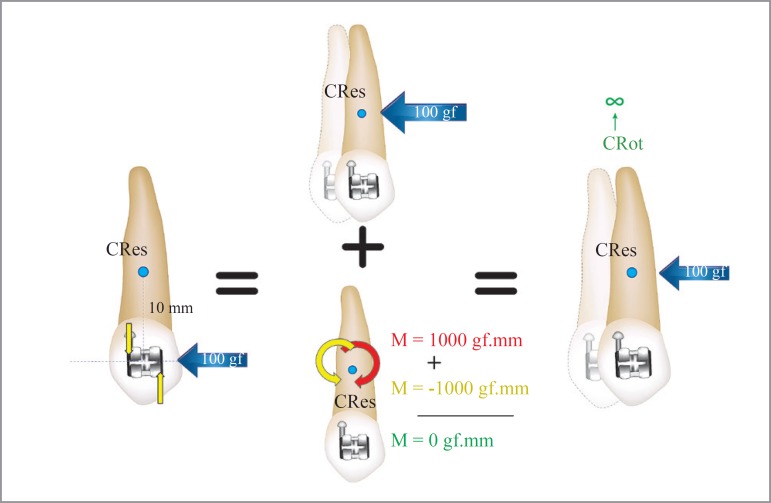
Example of an equivalent system. Canine subjected to a 100-g force (blue arrow) 10 mm away from its CRes, with a 1000-g.mm torque moving by translation. In red, the moment of force. In yellow, the moment of torque; and in green, the moment resulting from the CRes (Source: adapted from Almeida^31^).

Didactically speaking, to increase our understanding on the subject, it has been established that the distance from a canine bracket to its CRes (approximately ⅓ of the distance from the alveolar crest to the apex, more cervically) is of 10 mm (in normal clinical conditions). In these cases, if a horizontal force of 100 gf is applied to the bracket, the moment of force (M = F x d) equals 1000 gf.mm. Therefore, the tooth moves by uncontrolled tipping. However, biomechanically speaking, by means of an equivalent system of forces, it is possible to move a tooth by translation, even if force is applied to the brackets (Fig 5).^1,2,3^

In order to make such a movement, it is necessary to expel the moment of force, letting the horizontal force act. To this end, it is necessary to make a gable bend in order to create a torque in the bracket slot, producing a moment of -1000 gf.mm (in the opposite direction of the moment of force). That moment is known as moment of torque. Thus, the moment of force and the moment of torque are neutralized, and the horizontal force will be the only one acting in the tooth, promoting tooth translation (Fig 5). The ratio between the moment of torque (-1000 gf.mm) and the horizontal force (100 gf) is known as moment-to-force ratio (MF), and it determines the position of the CRot of the tooth, which, in this case, is 10/1 (absolute value). The MF ratio is the scientific basis to establish differential movements by means of an equivalent system of forces^1,2,3^ (Fig 5). Retraction of anterior teeth is a classic clinical example. Whenever orthodontic archwires exert forces onto brackets, anterior teeth undergo movement of retraction (horizontal force) and uncontrolled tipping (moment of force). In order to maintain correct axial tipping, it is necessary to apply a moment so as to oppose such tendency towards tipping (moment of torque) by means of incorporating vestibular torque of the crown (to brackets and/or archwires). Ideally, should there be a balance between the moment of torque and the moment of force (tendency towards uncontrolled tipping), a moment of retraction is established by translation.

**Figure 6:**
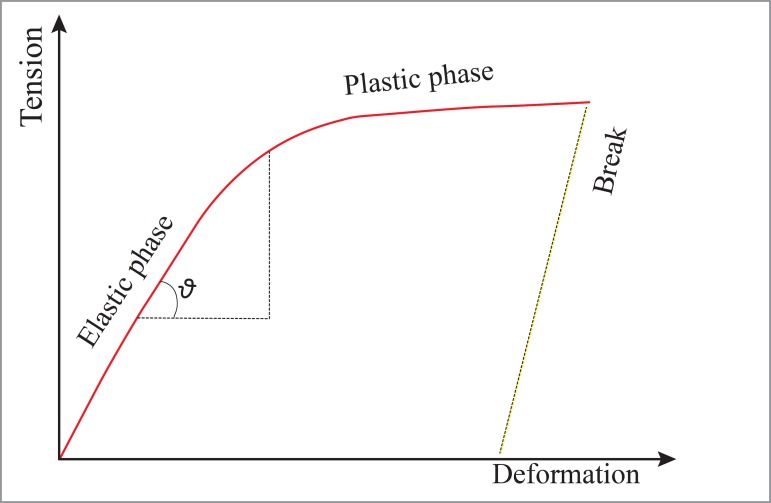
LF ratio of a wire is calculated by the slope of the line during its elastic phase.

The concept of load/deflection ratio (LF) is also key to understand SAT. Orthodontic wire tension-deformation charts show that such ratio is characterized by the slope of the line during the elastic phase of the wire, which corresponds to Hooke's law (Fig 6). Thus, clinically speaking, it is characterized by the amount of load (force) that is lost when the appliance is unloaded (deactivated).

In other words, the more bended a line is (for instance, a steal wire), the more force is lost for each millimeter of deactivation. On the other hand, beta-titanium wires (β-Ti) present a lower slope of the line, which results in a minor loss of force during deactivation, i.e., a lower LF ratio.

Low LF ratio wires are ideal for segmented mechanics, given that they tend to keep satisfactory levels of force during system deactivation, proving frequent follow-ups or continuous adjustments to be unecessary.^4,5^

Additionally, an increase in the distance between brackets, as a result of segmentation and the use of auxiliary tubes, increases the points of force application and allows more predictable mechanics to be carried out. Furthermore, increased distances allow springs to have their LF ratio decreased, which results in greater activations and a lower number of adjustments. Such increase in the distance between brackets allows accessories to be safely positioned and activations to be more safely performed in comparison to conventional mechanics.^1^

The advantage of not connecting adjacent brackets (with greater distance between them) can be used to selectively dissipate forces and moments. Differently from straight wire mechanics, in which reactive forces are dissipated around points of force application as a result of adjacently connecting the brackets, in SAT, the site where the reaction will be dissipated can be previously selected. Canine extrusion reactive forces and moments can, for instance, be dissipated in molars instead of incisors and bicuspids.^1^

In addition to that, the new metal alloys allowed excellent forces to be produced without changing the transverse section, in which case the concept of variable module was introduced to Orthodontics.

The advantages of the variable module include a better control of tooth movement as a result of opting for rectangular wires since the beginning of treatment, as well as reduction in the number of archwires used during mechanics and fabrication of appliances that produce horizontal force of less magnitude and low LF ratio. In this context, β-Ti wires have become indispensable for producing biological tooth movement through ideal forces, in which case these wires are chosen for SAT.^2^

β-Ti alloy, commercially known as TMA or titanium molybdenum alloy (Ormco Corporation, Glendora, USA), presents the properties of a "higher quality" wire, for instance: high springback properties, lower hardness values in comparison to steel, high formability and weldability with no reduction in resilience, and resistance to corrosion. It has higher springback properties in comparison to stainless steel and it is able to be deflected twice as much without permanent deformation. Additionally, it releases forces that correspond approximately to half of the forces released by steel alloys under similar activation, which causes its LF ratio to be approximately half of the stainless steel ratio.^6^

**Figure 7:**
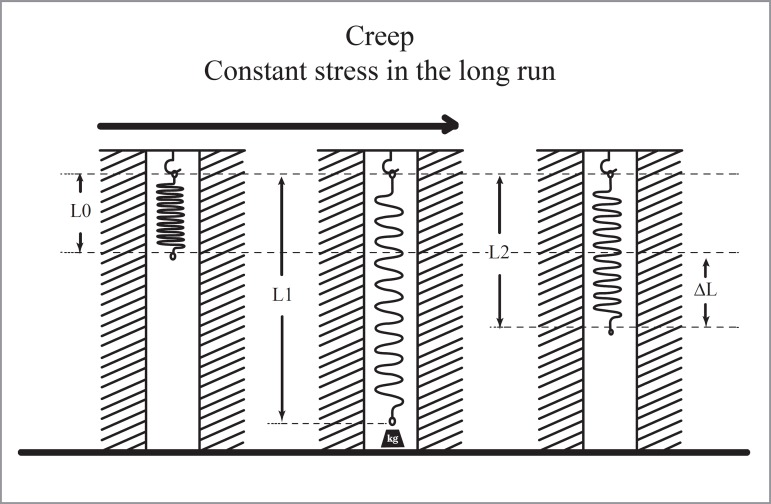
Example of creep (Source: adapted from Caldas et al^4^).

**Figure 8:**
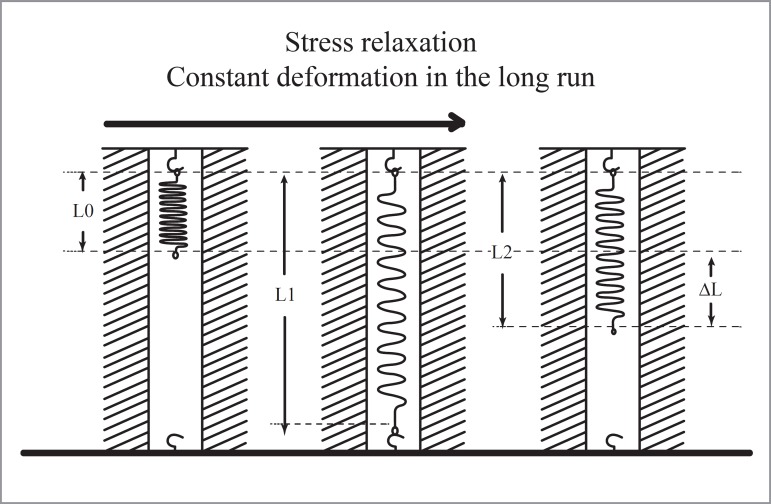
Example of stress relaxation.

However, the majority of clinicians do not remember that, in most types of material, plastic deformation occurs if tension exceeds its limit of elasticity,^5^ in other words, that plastic deformation is also time-dependent.^5,7^ Whenever a given material is constantly subjected to load or stress within its elastic limit, it may undergo progressive deformation known as creep.^5,7^ From a microscopic prospect, creep in metals results from displacements in the crystal structure of matter. Such microscopic phenomenon can be experimentally considered as an increase in deformation associated with constant stress (Fig 7) or an increase in stress associated with constant deformation (stress relaxation) (Fig 8). Creep depends on the intensity of stress and temperature, given that high tension and temperature favor displacement. In Engineering, creep in metals is only considered when the temperature accounts for at least 30% of the material melting point, given that structural components often undergo high tension.^5,7^

As for orthodontic springs, acute bends are generally employed to shape the wire. Such bends centralize stress, producing unstable spacing and displacement in the crystal structure of high tension sites.^5^ Orthodontists have tried to overcome such problem by thermally treating orthodontic stainless steel appliances so as to rearrange the crystal structure and, as a result, relieve residual tension.^8^ The Bauschinger^2^ effect is also another strategy commonly employed. It consists in overactivating the wire and simulating activations until the wire is properly shaped for applying the system of forces.

Such progressive deformation, as a result of structural stress relief, has been recently proved by mechanical trials conducted with β-Ti T-loop springs previously activated by bends, in which case reduction in force and moments of 15.5 and 17.15%, respectively, were observed within the first 24 hours. In clinical practice, applying an acute bend to shape an orthodontic appliance may cause the latter to loose and, as a consequence, change its system of forces under constant deformation. In these cases, the appliances must be overactivated or the acute bend must be replaced by curvature activation, i.e., gradual activation without significant sites of stress.^8^

Another fact that should be carefully assessed is the interchangeable use of β-Ti wires that are available on the market. Once the patent for the first commercial brand of β-Ti expired (TMA, OrmcoCo., Glendora, USA), the use of β-Ti alloys drastically expanded with a wide range of price and quality.^9^ In spite of the large number of brands available, only a few researches have been conducted to investigate the properties of this type of wire.^10,11,12^ Those researches compared the mechanical properties of β-Ti alloys by means of traction tests^11,12^ or 3-point flexural tests^10^ in straight wire segments. This may not demonstrate how different β-Ti alloys behave when bends are placed in the wires or when more elaborate designs are chosen (springs, loops, cantilevers, etc.). A recent research conducted with four different β-Ti wires (TMA [Ormco Co.], Beta Flexy [Orthometric Imp. Exp. Ltda, Marília/SP, Brazil], Beta III Wire [Morelli Ortodontia, Sorocaba/SP, Brazil] and Beta CNA [Ortho Organizers, INC., San Marcos, USA]) revealed that the wires produced different systems of force when used in the form of a more elaborate design (T-loop spring) due to the fact that each one of the wires responded differently when a bend was created. TMA and Beta CNA revealed more consistent MT ratios during trials. Such results do not mean that the other types of wire must not be clinically used; although they need to be employed with a different approach.^9^

Thus, SAT, as it has been previously stated, involves several mechanical concepts and particularities that must be well understood by those employing it. Its application is rather useful for performing a rational orthodontic treatment. However, it is recommended for specific cases, i.e., to treat specific conditions such as canine extrusion and retraction, deep bite correction, molars uprighting and occlusal plane correction.

## CANINES EXTRUSION

Maxillary canines are often impacted due to being the last teeth to irrupt in the oral cavity, having a complex eruption path, or due to the lack of space for appropriate positioning in the dental arch.^13^

In these cases, orthodontic traction may be carried out by means of SAT with the aid of a cantilever or a rectangular loop. As it has been previously mentioned, the use of mechanics with continuous wire, regardless of the type of bracket, produces several side effects on teeth adjacent to the canine. For this reason, it must be avoided. Therefore, by means of SAT, the desired movement is achieved when force is directly applied to the mal-positioned/impacted tooth, while reactive force is dissipated or controlled in the posterior anchorage unit as a result of an increase in the number of teeth joined to the segment and/or the use of an anchorage appliance.

**Figure 9:**
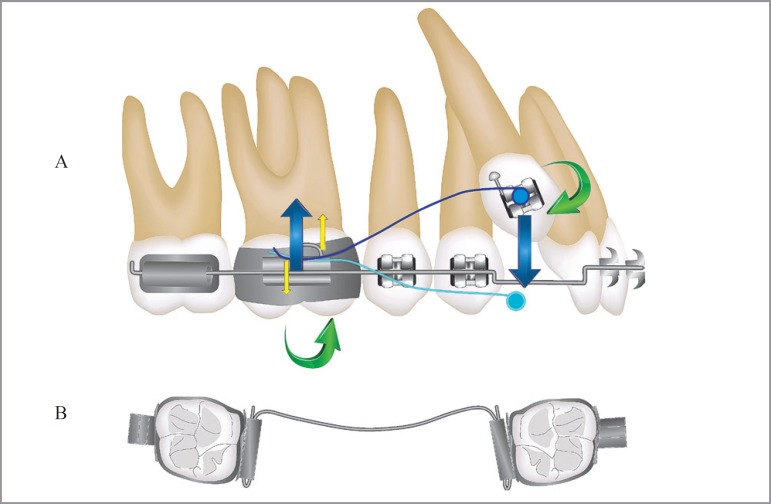
**A**) System of forces produced by a cantilever (statically determined system).This system also reveals a tendency towards palatalization of #13 with force going in the vestibular direction of CRes. **B**) Palatal bar used to control any undesired effects on the anchorage unit.

Clinically speaking, both appliances are feasible and effective. However, biomechanically speaking, they present peculiar characteristics that need to be well understood by the clinician. A cantilever can be described as a segment of a stainless steel 0.017 x 0.025-in wire or a β-Ti wire inserted inside a bracket or tube in the reactive member, tied to the opposite end by means of contact point in the active member (impacted or mal-positioned canine).Therefore, the wire is not inserted into the bracket slot where movement is desired. Should the force produced by an activated cantilever (ranging from 40 to 60 gf) be directed along the CRes of the tooth, it will produce a translatory movement. Should it bypass the CRes of the tooth (in most cases, it is applied to the crown), it will result in a rotational tendency of force due to the moment of force established (Fig 9).

Conversely, given that the wire has been inserted inside the bracket slot or tube, a force of equal intensity and opposite direction is observed in the reactive member. Similarly, a torque is produced inside the appliance when the moment arm of the activated cantilever is tied to the tooth to be moved. Such torque produces movement that, in association with force, must be restricted or reduced to nothing by reinforcing the anchorage of the reactive member (for instance, by using a palatal bar), so as to avoid side effects on the mechanics. Therefore, a cantilever is a statically determined system, characterized by force and moment of force in the active member, whereas it is characterized by force of similar intensity, but opposite direction, and moment of torque in the reactive member. Such system provides a system of forces that can be easily visualized and predicted during the process of deactivation (Fig 9).

**Figure 10:**
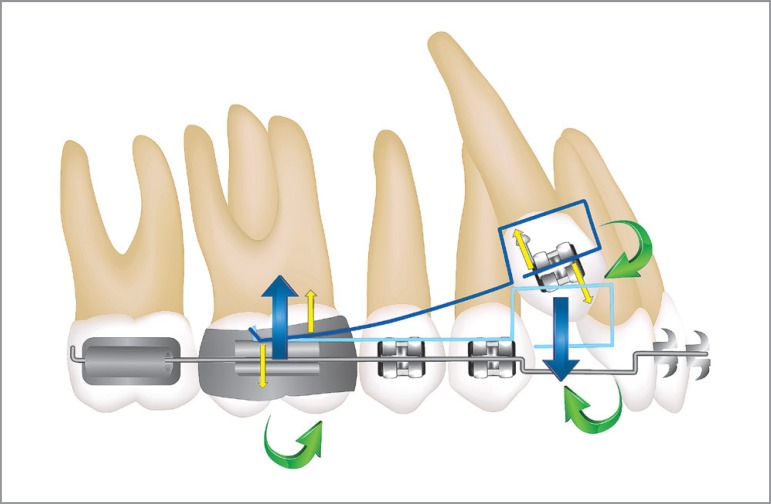
System of forces produced by a rectangular loop (statically undetermined system). The activated rectangular loop (light blue) must be adjusted so as to determine the final position of the tooth. When inserted into the slot (dark blue), its deactivation will move the tooth in the previously established position.

A rectangular loop is recommended for tridimensional control of a tooth with more severe positioning anomalies. It may be fabricated with stainless steel 0.017 x 0.025-in wire or β-Ti wire between 6 and 7 mm in the cerviccal-occlusal direction, and between 8 and 10 mm in the mesiodistal direction.^13^ Unlike a cantilever, a rectangular loop is inserted inside the bracket slot of an active member. Therefore, in this case, in addition to force (moment of force), a moment of torque is also established as a result of inserting the wire inside the slot. In the reactive member, the system is similar to a cantilever. This system is statically undetermined, given that during deactivation, the system of forces may be little predictable due to changes in tooth positioning (Fig 10).

Thus, it is recommended that a cantilever be used in buccally or lingually placed impacted canines that need to be lingually or labially repositioned, respectively. A cantilever allows a system of forces to be applied in the desired direction, which may be determined by the method of preactivation. A cantilever may be replaced by a rectangular loop, provided that the crown of the canine is apparent and its movement may be three-dimensionally controlled until correct positioning is achieved in the dental arch.^13^

## CANINE RETRACTION

Canine retraction for resolution of previous crowding is a clinical condition that largely favors the use of SAT before straight wires are used. Should the orthodontist choose to perform mechanics with straight wires avoiding buccal tipping of incisors, straight wires will be progressively used without including the incisors in the arch. Canine retraction begins with an E-arch (rectangular steel wire). Another option would be passive E-arch in the posterior region and canine, which would allow retraction since the first month of treatment.

**Figure 11:**
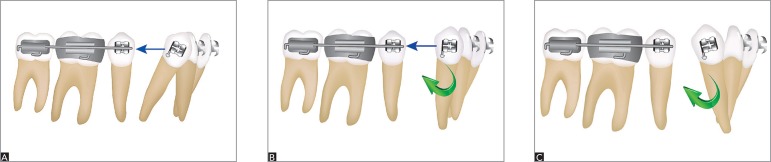
**A**) Mesially tipped canine and the initial need for horizontal force, only. (**B**) Upright canine must undergo translation movement, i.e., a combination of horizontal force and moment. (**C**) Distal tipping of canine initially suggests the application of moment for root movement.

Another relevant point is mesiodistal tipping of canines, in which case three situations should be considered: canine mesially tipped with the apex more distally placed than the cuspid tip; upright canine with root apex and cuspid tip virtually occupying the same position in the mesiodistal direction; distally tipped canine with the apex more mesially placed in relation to its cuspid (Fig 11). Using straight wires for cases of mesial tipping and upright teeth favor the resolution of crowding and canine retraction (Figs 11A and 11B). Placing the straight wire with the canine mesially tipped favors distal movement of the crown, which results in diastema between the canine and the lateral incisor, thus favoring the resolution of crowding (Fig 11A).

Conversely, upright canines do not favor the opening of diastema, but it is probable that, in this case, the onset of retraction will happen more quickly (Fig 11B).

On the other hand, in case of distal tipping of canines (Fig 11C), alignment and leveling with straight wires may worsen the condition of crowding, in which case there is a tendency towards mesial movement of the crown. The use of elastomeric chain or springs for canine bracket in the posterior region is also contraindicated to avoid mesialization of the crown, given that these devices favor distal tipping of the crown. Additionally, extrusive and undesired forces will be incorporated in the region of incisors during alignment.

Thus, it is recommended that segmented mechanics be used with reactive members and pre-activated T-loop springs, and that it be horizontally activated in accordance with the mesiodistal tipping of canines. For instance, in case of mesial tipping, the T-loop spring may be horizontally activated until canine uprighting is achieved. As for upright canines, a standard T-loop spring is used, i.e., with pre-activations (moment) and horizontal activation, producing translatory movements. In case of distal tipping, pre-activations (moment) may be initially used. After distal movement of canine roots is produced and uprighting is achieved, standard T-loop spring activation begins. Anti-rotation bends must be considered to overcome the tendency towards rotation of canines, also treated according to the position of canines in the dental arch. A complete description of T-loop spring biomechanics is found in publications by Martins et al.^1,4,8,9,14-17^

## DEEP BITE

Deep bite is a multifactorial vertical malocclusion that needs a differential diagnosis comprising facial, dental and cephalometric data as well as data on the vertical positioning of incisors at rest, smile and speech.^18^

It may be treated by extrusion of posterior teeth, intrusion of maxillary and/or mandibular incisors or a combination of procedures.^18^

**Figure 12:**
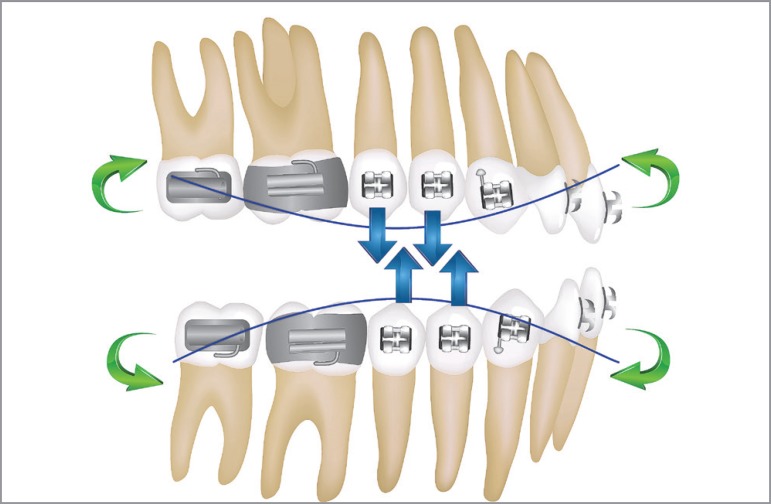
Summary of the mechanical effects of arches used to manipulate the curve of Spee.

Intrusion of incisors was considered for many years as a complex orthodontic movement. In fact, it is a technical movement that must be carefully planned. The use of straight wires hardly produce pure intrusion of incisors, instead, it leads to extrusion of posterior teeth. In other conditions, such as in appropriate anterior overbite, alignment and leveling achieved with straight wires may lead to deep bite, given that canines with severe root mesial tipping (Fig 11C) during correction cause anterior overbite to deepen. Following this line of reasoning, using archwires to level the curve of Spee (excessive in the maxillary arch and reverse in the mandibular arch), regardless of the type of alloy, will promote extrusion of posterior teeth, especially of premolars, followed by pseudo-intrusion of incisors (more buccal tipping than pure intrusion) (Fig 12).^18^

A systematic review conducted in 2005 concluded that the movement of intrusion is feasible and more easily achieved in the mandibular arch than in the maxillary arch. The study revealed that the segmented arch technique is most commonly employed for intrusion of incisors, with 1.5 mm of intrusion of maxillary incisors and 1.9 mm of mandibular incisors.^19^

**Figure 13:**
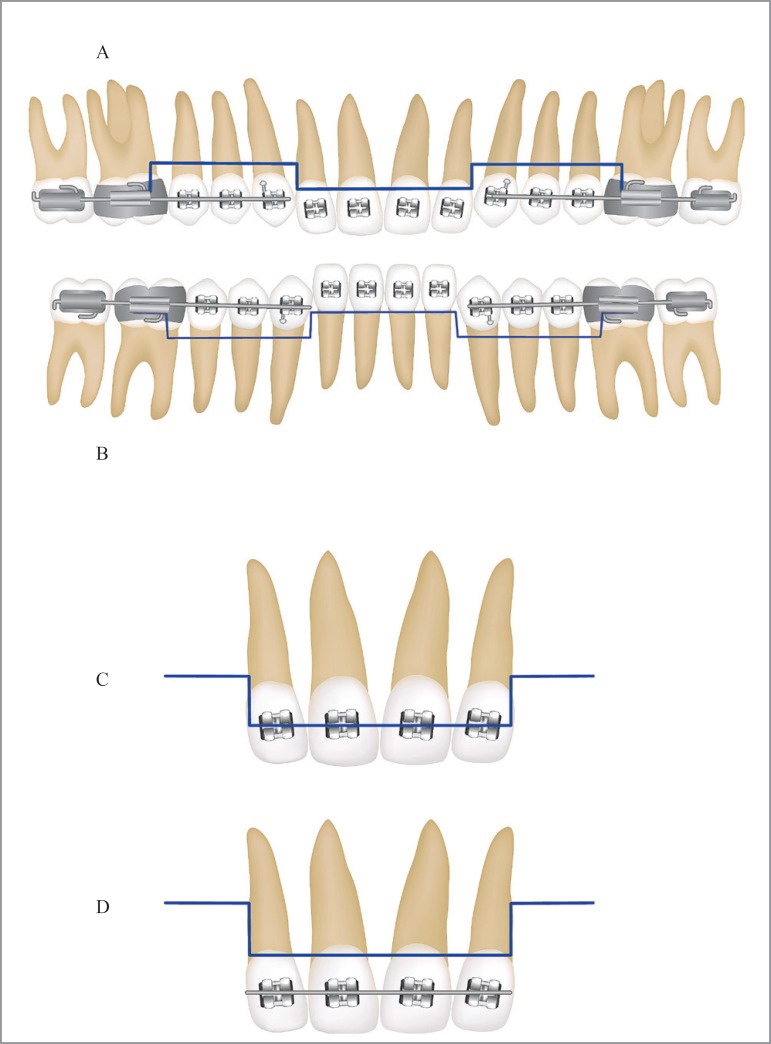
Illustration of the straight wire intrusion system. (**A**) maxillary intrusion arch; (**B**) mandibular arch; (**C**) bracket fitting; and (**D**) fitting in another segment of wire.

Intrusion of anterior teeth can be basically achieved by means of two types of mechanics: straight wire appliance and three-piece arch appliance. The former consists of a segment of wire placed along the dental arch and, in the anterior region, placed on the teeth by directly fitting into the bracket slots (Ricketts arch) (Figs 13A, 13B and 13C) or by being tied to another anterior segment (Burstone arch) (Fig 13D).

**Figure 14:**
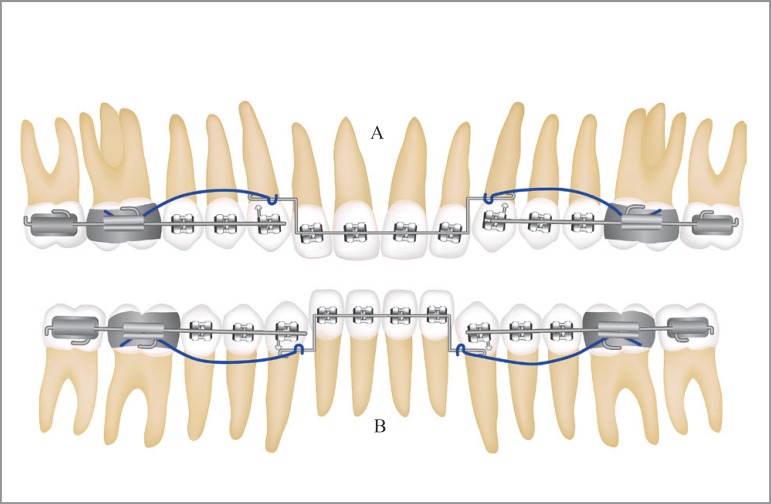
Illustration of the three-piece arch appliance intrusion system. (**A**) maxillary arch; (**B**) mandibular arch.

As for the three-piece arch appliance, two intrusion cantilevers are fabricated (one for each side) and inserted into another segment in the anterior region (Fig 14). Using a segment of wire in the anterior region, isolating the area, is a much more predictable and understandable alternative. Should the intrusion arch be directly inserted into the anterior bracket slots (Fig 13C), undesired torques may be incorporated, and a more complex and undetermined mechanical system may be produced.

**Figure 15:**
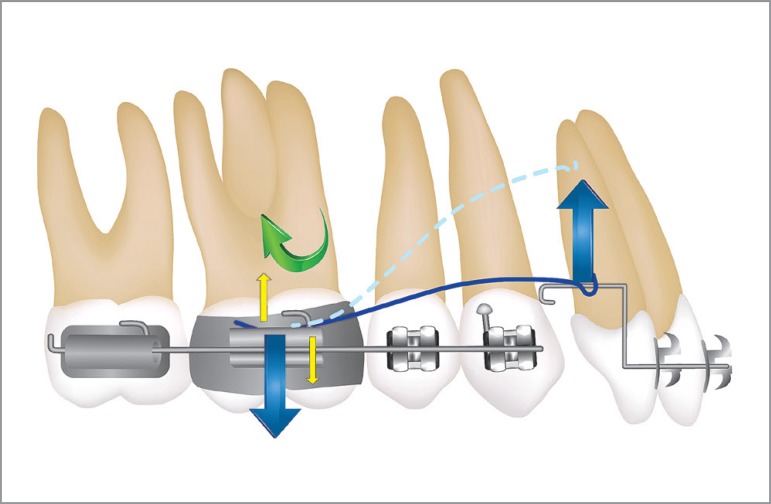
Intrusion system with the forces and moments produced.

Regardless of the type of fitting, good anchorage control must be achieved due to the forces and, especially, the moments created by the system of intrusion (Fig 15). The largest number of posterior teeth must be incorporated into posterior segments, with lingual or palatal arches used to gather posterior segments into one single anchorage segment.^2^ Additionally, the use of a high-pull headgear and short power arm angulated upwards is also recommended to balance the adverse effects of this mechanics. Likewise, the use of mini-implants or miniplates is also recommended.

According to Burstone, the key to success in intrusion is controlling the system of forces. Light, constant forces must be employed, with the point of application and direction of force carefully assessed. The magnitude of force must range from 10 to 15 gf per incisor. To calculate the total force, the teeth that will potentially undergo intrusion must be added up and the corresponding load must be applied. The magnitude of force is of paramount importance to yield satisfactory clinical results. For this reason, it is recommended that precision dynamometers be used to calculate the appropriate load.^2^

Researches have been conducted on the point of force application in order to provide appropriate intrusion of maxillary incisors. The results yielded by these studies led to clinical assertions that oftentimes result in errors. When all four incisors need to be intruded with maintenance of axial inclination (without moment production), intrusive force must be applied near the distal surface of lateral incisors. In order to produce intrusion and buccal tipping of incisors, force must be mesially applied to lateral incisors, whereas for intrusion with retroclination, load must be distally applied to canines.^20^

**Figure 16:**
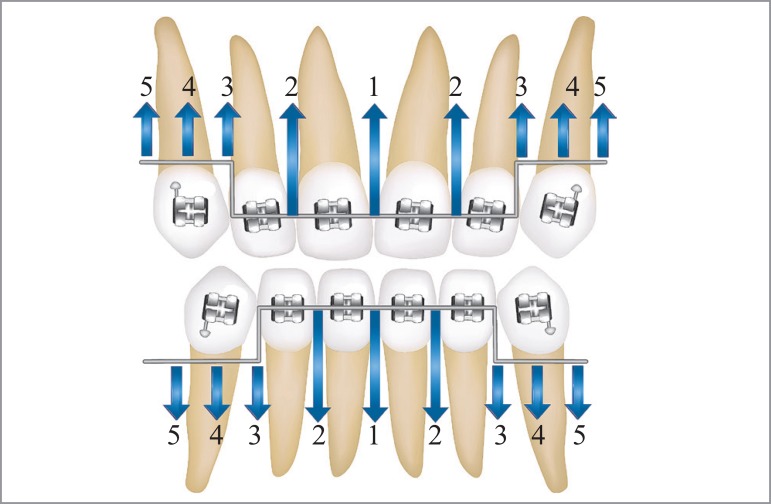
Different points of force application and mechanical effects on incisors: 1, 2 and 3 = intrusion + buccal inclination; 4 = intrusion; and 5 = intrusion + retroclination.

Santos-Pinto^21^ also suggests other points of force application, which vary between pure intrusion and intrusion associated with buccal tipping or retroclination, according to the objectives of treatment (Fig 16).

It is worth mentioning that these points of force application must be adapted to the conditions of each clinical case. Depending on the degree of axial inclination of incisors, such points may be more anteriolly or posteriolly displaced.

**Figure 17:**
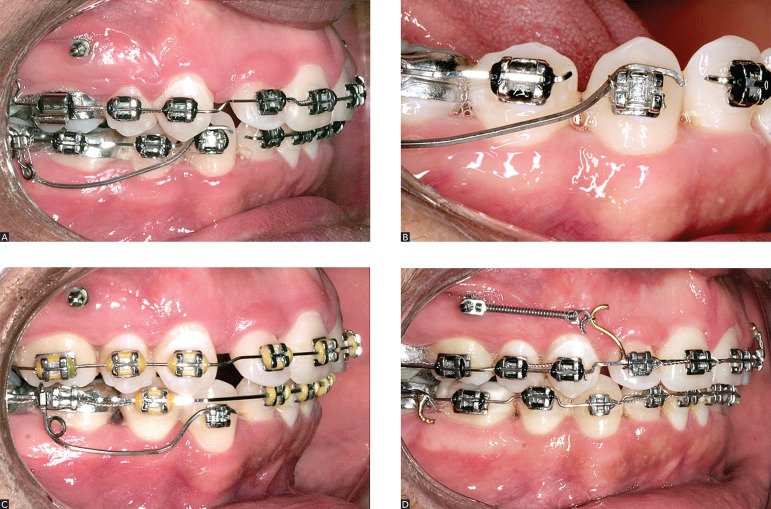
Clinical illustration of a cantilever used for canine intrusion.

Some clinical conditions require not only intrusion of incisors, but also intrusion of canines, in which case it is recommended to begin deep bite correction by canines followed by incisors. Figure 17, for instance, shows the use of a three-piece arch appliance, with two segments posteriorly placed and one segment placed on the incisors without including the canines. Two cantilevers were fabricated with β-Ti wire for intrusion of canines on both sides. Subsequently, when canines are overcorrected or on the same occlusal plane of posterior teeth, they are incorporated to the poster anchorage unit, after which intrusion of incisors is performed.

Today, intrusion of maxillary incisors has been rarely used as the only resource to treat deep bite, given that exposure of maxillary teeth and a narrow section of gingival tissue yield more esthetic results, giving the patient a younger appearance.^18^ Slightly extruded maxillary incisors are generally more esthetic than slightly intruded teeth.^22^ On the other hand, in very specific cases, intrusion of maxillary incisors is well recommended.

**Figure 18:**
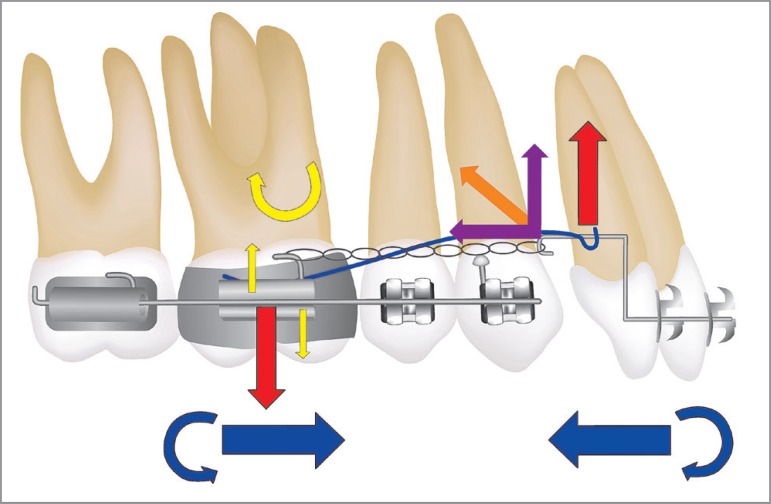
Mechanical illustration of forces and moments in the segmented arch technique employed for simultaneous retraction and intrusion of incisors.

In addition to the aforementioned procedures, intrusion and simultaneous retraction of maxillary incisors are highly recommended not only for antero-superior intrusion, but also for retraction, effectively controlling axial inclination of those teeth.^23^ In these cases, in addition to the three-piece arch appliance, retraction appliances, such as elastomeric chain or springs, are also used, thus incorporating new forces and moments into the system (Fig 18). Similarly to the mechanics described in Fig 15, good anchorage control must be implemented so as to overcome the side effects of the mechanics.

**Figure 19:**
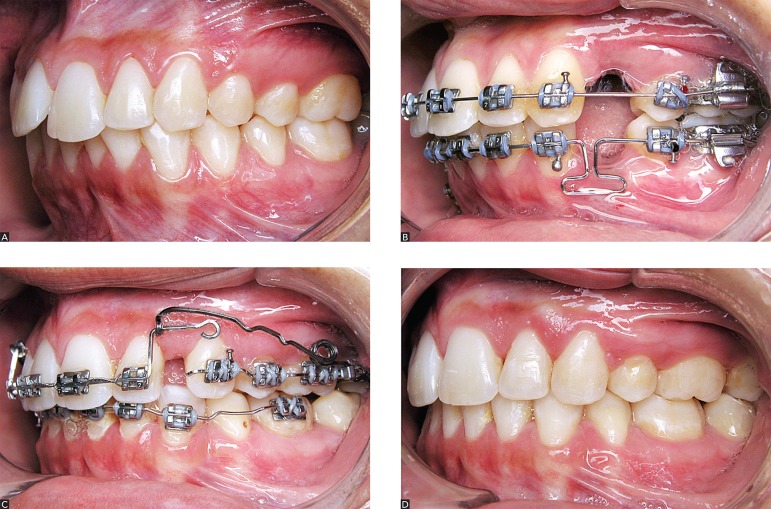
Clinical illustration of SAT used for simultaneous retraction and intrusion of incisors:(**A**) initial case; (**B**) during maxillary alignment and leveling; (**C**) deep bite after alignment and leveling completion, and partial retraction of canines; (**D**) final result after placement carried out by means of the segmented arch technique.

As it has been previously mentioned, in many clinical situations, alignment and leveling achieved by means of straight wires may cause deep bite. Furthermore, in case of extraction of first bicuspids, partial retraction of canines with straight wires also increases deep bite. Figure 19 shows how deep bite was achieved after alignment, leveling and partial retraction of maxillary canines. In these cases, retraction of incisors becomes a difficult task to be achieved due to the lack of overbite and overjet necessary for retraction. In this example (Fig 19C), the arch of intrusion and simultaneous retraction was used for retraction of incisors with greater control of axial inclination without necessarily promoting real tipping. At treatment completion (Fig 19D), we observed that retraction was achieved with correct inclination of incisors, whereas anterior overbite remained unchanged, preventing potential deleterious impact on the degree of teeth exposure at smile, rest and speech.

## MOLAR UPRIGHTING

Molar uprighting is widely employed in cases of early loss of deciduous teeth followed by deleterious dental migration in young patients and in orthodontic treatment of adult patients with tooth loss. In these cases, molar uprighting is generally associated with extrusion of antagonist teeth, reduction in edentulous space, bone dehiscence in the mesial surface of tipped molars, gingival recession of tipped molars, early contact in centric relation and occlusal interference on excursion of the mandible.^24^ With regard to integrated planning, clinicians must decide whether the tooth subject to uprighting will undergo movement for space closure, opening of space for prosthetic rehabilitation or implant placement.

Mesial movement of molars may be rendered difficult due to the following: alveolar bone resorption resulting from tooth loss, which causes the molar mesial bone to become too thin; unfavorable root morphology for movement of lower molars; greater mandibular bone density in relation to the maxilla; and thin buccolingual bone thickness from distal to mesial in the mandibular arch.

**Figure 20:**
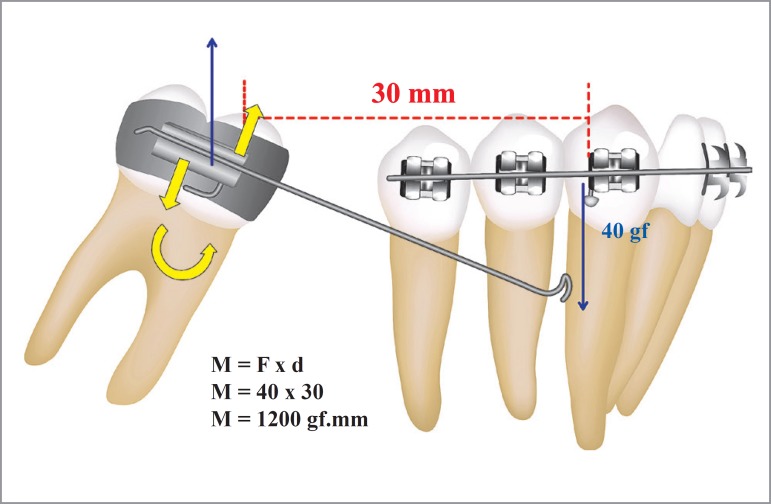
Illustration showing the moment produced by the cantilever used for molar uprighting.

Using straight wires to upright tipped molars is considered unfeasible, given that, in these cases, there is a strong tendency towards extrusion of molars, especially due to the short distance between brackets. Additionally, incorporating a T-loop spring into the arch will lead to extrusion of premolars.^24^ A cantilever, extended up to the anterior region, may be used to reduce the effects of extrusion on molars. Researches have proved a moment of 1200 gf.mm to be appropriate for molar uprighting.^25,26^ Should a 30-mm cantilever be used, an activation of 40 gf is enough for molar uprighting (Fig 20), in which case 40 gf corresponds to intrusive forces in the anterior region and extrusive forces in the region of molar teeth. Mesocephalic or brachycephalic patients are able to eliminate or reduce this effect of extrusion by their own muscular pattern; however, additional studies are warranted to further investigate this topic. Another aspect that should be highlighted is whether or not the tooth subject to uprighting allows attrition, such as extensive restoration, on its occlusal surface. In other words, whether it is a tooth that will become a metal-ceramic crown that allows extrusion as it will undergo attrition.

**Figure 21:**
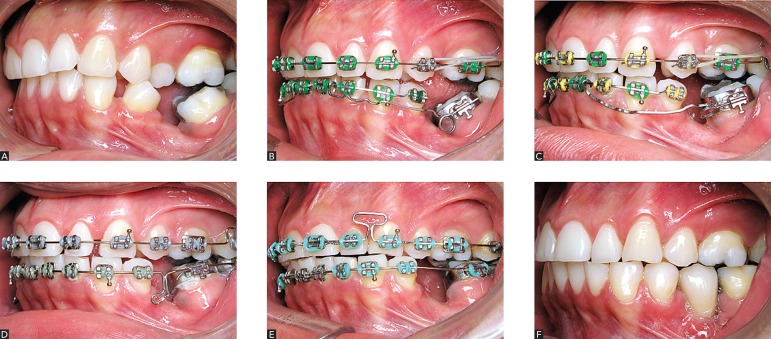
Clinical illustration of a cantilever used for uprighting tooth #37.

Figure 21 shows a clinical case of side effects on occlusion resulting from loss of first molar and lower second premolar. The second molar was not included in the straight archwire during alignment and leveling. Subsequently, a β-Ti cantilever was used for two purposes: 1) molar uprighting and space opening for implant rehabilitation; and 2) correction of the occlusal plane in the anterior segment as a result of installing the cantilever in the midline, thus producing intrusive forces in this area (Figs 21B, 21C). With the molar near the occlusal plane, straight wires were used to go on with treatment and reach completion (Figs 21D, 21E, 21F).

**Figure 22:**
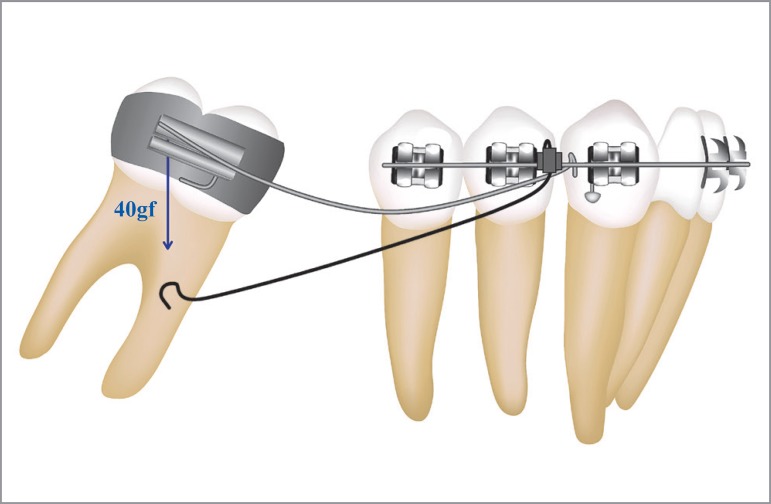
Illustration showing the effects of intrusion caused by activation of a second cantilever inserted into the cross tube, neutralizing the extrusive effects produced by the first cantilever.

**Figure 23:**
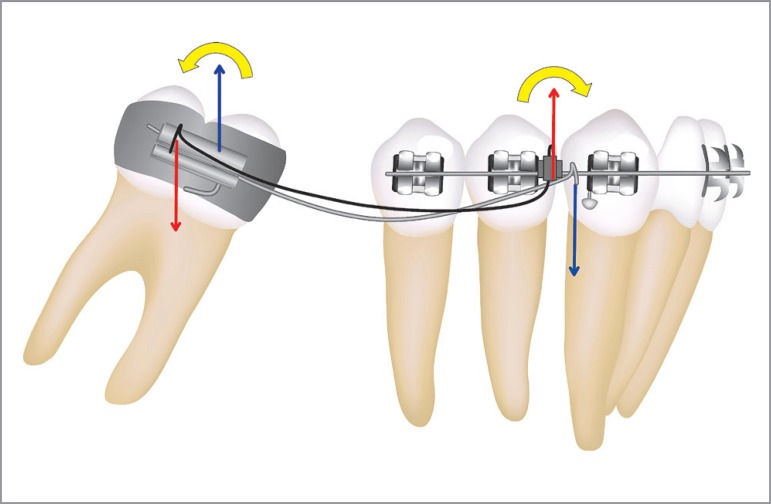
Illustration showing the neutralization of vertical effects on molar subject to uprighting.

In cases in which molar extrusion is impossible, springs or cantilevers supported by mini-implants can be used with the vector line of force passing under the molar CRes, so as to produce a resultant force that intrudes the molar. Another option to eliminate extrusion force is to use a double cantilever,^27^ in which case one cantilever is used as in conventional molar uprighting, while the other is activated to produce intrusive forces on the molar. Thus, the effect of extrusion produced by the conventional cantilever is neutralized by the second cantilever. One disadvantage of this option is patient's discomfort caused by the concurrent use of two devices (Figs 22, 23).

## OCCLUSAL PLANE CORRECTION

Occlusal plane inclination is a daunting challenge faced by orthodontic treatment. It is mainly caused by dental issues such as loss and/or ankylosis of antagonist teeth, deleterious oral habits, inappropriate orthodontic bonding leading to asymmetric dental alignment, skeletal disharmony or a combination of factors.^28^

Most cases of skeletal facial asymmetry present significant changes in maxillary and/or mandibular occlusal plane. TJD-related pathologies (rheumatoid arthritis, condylar hyperplasia, osteochondroma, condylar resorption, and others) and craniofacial syndromes/deformities are the most common primary etiological factors of facial asymmetry that impairs the occlusal plane. In fact, abnormal mandibular impairment is initially observed in growing and non-growing patients.

However, to compensate such changes, the maxillary occlusal plane is also impaired so as to establish a balance between the mandibular changes.^28,29^

Treatment of those pathologies depends on the cause and degree of severity of the change. In cases of significant facial alterations, dental correction may be achieved by mini-implants or miniplates; however, no improvements in facial esthetics will be observed. Thus, an approach combining orthognathic surgery and orthodontic treatment is recommended. Nevertheless, in borderline cases in which patient's chief complaint is not facial, orthodontic approach alone is employed with considerable forseability.^29^

**Figure 24:**
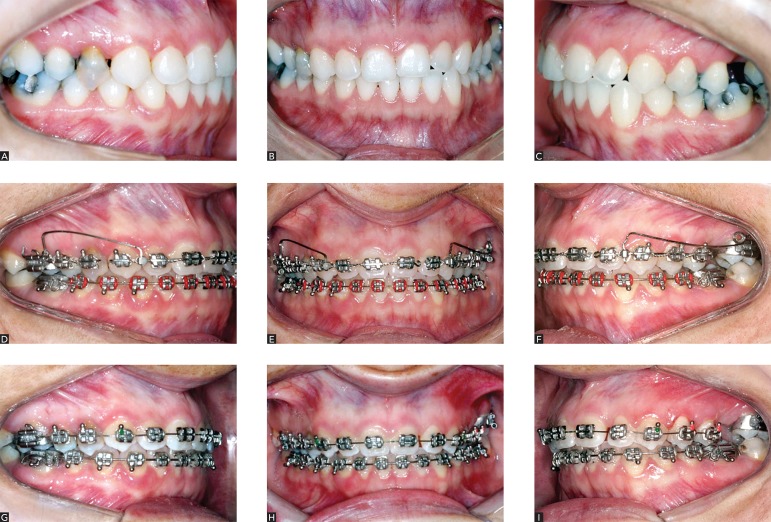
Clinical illustration showing the use of two asymmetric cantilevers. A, B, C) Initial case; D, E, F) Intrusion on the right side and extrusion on the left side for correction of the occlusion plane; G, H, I) After mechanics was applied.

Mini-implants became popular and allowed those issues to be addressed in a more predictable manner, yielding excellent results.^30^ Nevertheless, other biomechanical procedures that do not require skeletal anchorage can be employed to treat occlusal plane inclination. The rational use of biomechanics by means of SAT and asymmetric cantilevers proves to be a feasible option. As shown in Figure 24A, the patient, whose chief complaint was having an asymmetric smile, presents occlusal plane inclination. Due to the absence of facial complaints, she was advised to undergo dental treatment, only, which would be performed with asymmetric cantilevers.

Initially, alignment and leveling were carried out in association with anchorage preparation of the reactive member by means of a palatal bar.

The 0.019 x 0.025-in wire was achieved and the arch was segmented into three pieces (from #16 to #14, from #13 to #21 and from #22 to #25). Initial alignment and leveling did not include tooth #27 which was coupled with #16 by a palatal bar.

The segment going from #13 to #21 underwent a clockwise movement, with the point of force application between #13 and #12, where greater intrusion was observed. A 70-g force was applied by a cantilever made of 0.017 x 0.025-in β-Ti wire (Fig 24B).

The segment going from #22 to #25 underwent an anti-clockwise movement, with the point of force application between #22 and #23. Extrusive force was applied by a cantilever made of 0.017 x 0.025-in steel wire. In order to achieve flexibility and decrease the LF ratio, a helix was placed in the wire with a force of (Figs 24D, 24E, 24F).

The side effects produced by the mechanics were balanced by the palatal bar reinforced with the use of a steel stabilization 0.019 x 0.025-in archwire placed from #16 to 14 on the right side. Treatment lasted for three months, followed by rebonding of brackets and new procedures of alignment and leveling (Figs 24G, 24H, 24I).

## FINAL CONSIDERATIONS

Today, orthodontists must keep distance from conceptual dogma and be open to new information that contributes to perform orthodontic treatment as effectively as possible. Knowing how to deal with potential side effects is essential for orthodontic therapy, given that such effects may hinder treatment and, in many cases, cause lost of control.

In addition to the aforementioned situations, the segmented mechanics may be employed in many other branches of Orthodontics, namely: anterior retraction, use of palatal bar and removable lingual arch (correction of rotation, distalization, arch expansion or contraction), extrusion of incisors, etc. Therefore, understanding the principles behind scientific biomechanics is crucial to yield great clinical results with as little side effects as possible. In this context, SAT proves to be essential as an auxiliary resource that aids the straight wire technique in several clinical situations.
